# Supplementary material for: Causal Relationship between Aging and Anorexia Nervosa: A White-Matter-Microstructure-Mediated Mendelian Randomization Analysis
Source: Biomedicines. 2024 Aug 16;12(8):1874. doi: 10.3390/biomedicines12081874 (PMC11351342; doi:10.3390/biomedicines12081874)
Supplement: Supplementary file 1 [file biomedicines-12-01874-s001.zip › Supplementary Figure/Figure S4/Singe SNP plot of MR analysis for TL to ubm-b-1796.pdf]

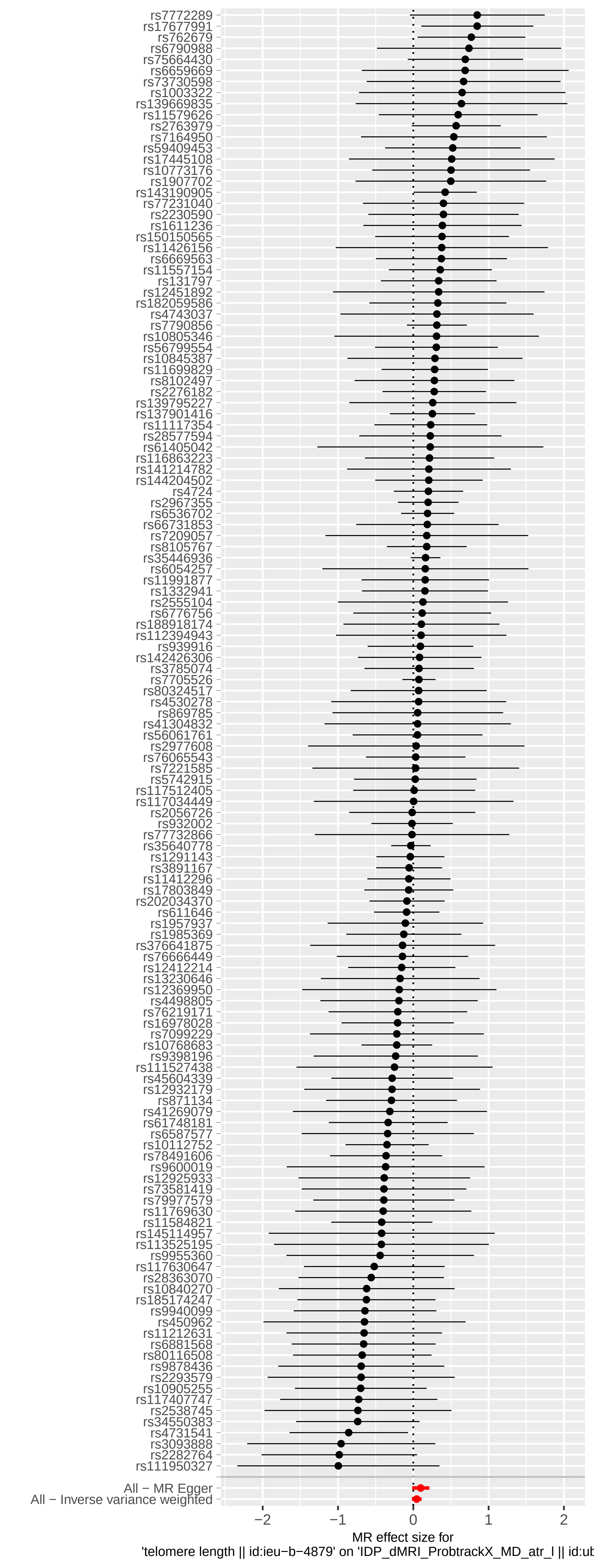

rs7772289
rs17677991
rs762679
rs6790988
rs75664430
rs6659669
rs73730598
rs1003322
rs139669835
rs11579626
rs2763979
rs7164950
rs59409453
rs17445108
rs10773176
rs1907702
rs143190905
rs77231040
rs2230590
rs1611236
rs150150565
rs11426156
rs6669563
rs11557154
rs131797
rs12451892
rs182059586
rs4743037
rs7790856
rs10805346
rs56799554
rs10845387
rs11699829
rs8102497
rs2276182
rs139795227
rs137901416
rs11117354
rs28577594
rs61405042
rs116863223
rs141214782
rs144204502
rs4724
rs2967355
rs6536702
rs66731853
rs7209057
rs8105767
rs35446936
rs6054257
rs11991877
rs1332941
rs2555104
rs6776756
rs188918174
rs112394943
rs939916
rs142426306
rs3785074
rs7705526
rs80324517
rs4530278
rs869785
rs41304832
rs56061761
rs2977608
rs76065543
rs7221585
rs5742915
rs117512405
rs117034449
rs2056726
rs932002
rs77732866
rs35640778
rs1291143
rs3891167
rs11412296
rs17803849
rs202034370
rs611646
rs1957937
rs1985369
rs376641875
rs76666449
rs12412214
rs13230646
rs12369950
rs4498805
rs76219171
rs16978028
rs7099229
rs10768683
rs9398196
rs111527438
rs45604339
rs12932179
rs871134
rs41269079
rs61748181
rs6587577
rs10112752
rs78491606
rs9600019
rs12925933
rs73581419
rs79977579
rs11769630
rs11584821
rs145114957
rs113525195
rs9955360
rs117630647
rs28363070
rs10840270
rs185174247
rs9940099
rs450962
rs11212631
rs6881568
rs80116508
rs9878436
rs2293579
rs10905255
rs117407747
rs2538745
rs34550383
rs4731541
rs3093888
rs2282764
rs111950327
All − MR Egger
All − Inverse variance weighted
−2
−1
0
1
2
MR effect size for
'telomere length || id:ieu-b-4879' on 'IDP_dMRI_ProbtrackX_MD_atr_l || id:ub
